# Africa has unique and urgent barriers to cleft care: lessons from practitioners at the Pan-African Congress on Cleft Lip and Palate

**Published:** 2012-05-30

**Authors:** Oluwaseun Adetayo, Rachel Ford, Mark Martin

**Affiliations:** 1 Department of Plastic Surgery, Loma Linda University, 11175 Anderson Street, Suite 21126, Loma Linda, CA, USA

**Keywords:** Cleft care, cleft lip, cleft Palate, craniofacial cleft, Nigeria, Africa

## Abstract

**Background:**

The goals of this study were to delineate the protocols employed for managing patients with cleft lip and palate deformities, delineate the challenges facing practitioners and patients, and to determine the patient and physician barriers to cleft care delivery in the region.

**Methods:**

Survey questionnaires were administered to practitioners attending the second Pan-African Congress on Cleft Lip and Palate (PACCLIP), which took place in Ibadan, Nigeria, West Africa from February 4-7, 2007. The conference included 225 participants, representing 17 African countries

**Results:**

Protocols for repair of cleft lip and palate deformities were varied, with Millard's and von Langenbeck's techniques being the preferred approach for the management of cleft lip and palate deformities, respectively. A large proportion of providers have limited access to core cleft care supporting teams, especially speech language pathologists, orthodontists, and audiologists. Several challenging barriers to cleft care were also identified at both the institutional and individual levels and are reported.

**Conclusion:**

Geographic separation in Africa presents a similar challenge due to isolationism as it does to surgeons in Europe. Specific to Africa are the increased barriers to care, and economic and financial hardship at various levels. A focus on funding, team building, infrastructural support, and patient education appear to be crucial in improving the care and lives of children with facial clefts in Africa.

## Background

The estimated prevalence of cleft lip and/or palate in the Nigerian population is 0.4:1000 [1]. Several studies have reported on cleft lip and palate abnormalities, but these have been limited to certain centers, cities, or geographic areas [2–6]. The interplay of the environment, genetics, cultural and religious beliefs, lack of education, financial constraints, low life expectancy, poverty and emigration of specialists outside the continent have been reported as factors contributing to the impediment of progress in cleft care [7–10]. The interplay of these factors, impact of negative perceptions of cleft patients, and the role of inadequate treatment and rehabilitation have also impeded cleft care. Some authors recommend placing emphasis on the need for public enlightenment programs and financial assistance to patients [10, 11] in order to alleviate some of these obstacles.

This study set out to directly elucidate these factors and their impact on providers offering cleft care by surveying African cleft care providers attending the second annual Pan-African Congress on Cleft Lip and Palate. The aim was to capture a diverse group of practitioners that were representative of the region being examined, thereby overcoming some of the limitations constraining past studies that were restricted to certain centers, cities, or geographic areas on the continent.

## Methods

We conducted a survey of participants at the second Pan-African Congress on Cleft Lip and Palate, which took place from February 4-7, 2007 in Ibadan, Nigeria. In 2006, Loma Linda University and Adventist Health International partnered with The Smile Train in coordinating the first-ever Pan-African Congress on Cleft Lip and Palate. The venue was set in Nigeria, West Africa, which has the largest population in the continent and is ranked ninth in the world for cleft births [11]. The conference included over 225 participants, representing 17 African countries ranging from surgeons to medical students. There were 140 surgeons in the fields of Plastic, Oral and Maxillofacial, and Ear, Nose, and Throat Surgeries. The remaining participants were comprised of nurses, dentists, residents, social workers, speech therapists, dental technologists, and medical students. There were 68 responders to the survey and the data collected is analyzed and presented.

Data was collected regarding percentage of isolated and combined cleft lip and palate cases encountered, percentage of patients seen with cleft abnormalities, their ages at presentation, techniques employed for repair, and cleft care team compositions. Information was also collected regarding physician and patient barriers to care, patient volume, and study demographics. The study also examined providers′ perspectives on deterrents to care, problems with access, and their perspectives on critical areas for improvement. Institutional Review Board approval was obtained based on our institution's protocol.

## Results

### Timing of Repair

**Figure 1:**
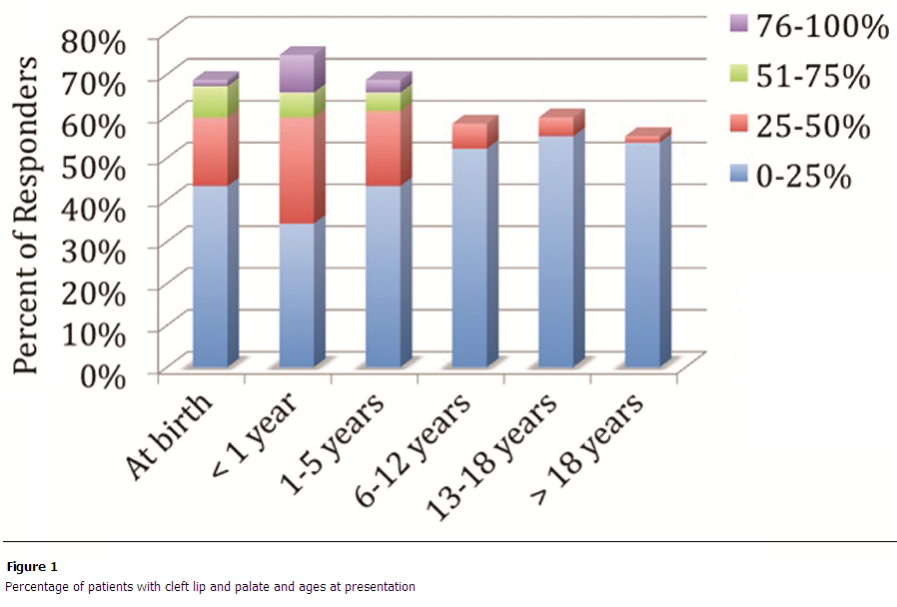
Percentage of patients with cleft lip and palate and ages at presentation

**Figure 2:**
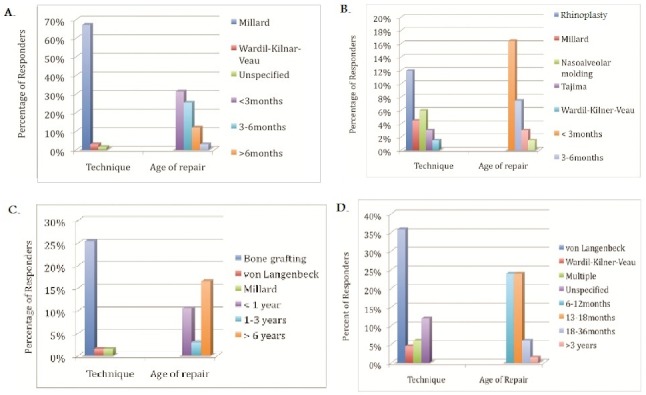
Protocols for repair of complete unilateral cleft lip and palate seen during the neonatal period for: A. cleft lip B. cleft nasal deformity C. alveolar deformity D. hard palate

**Figure 3:**
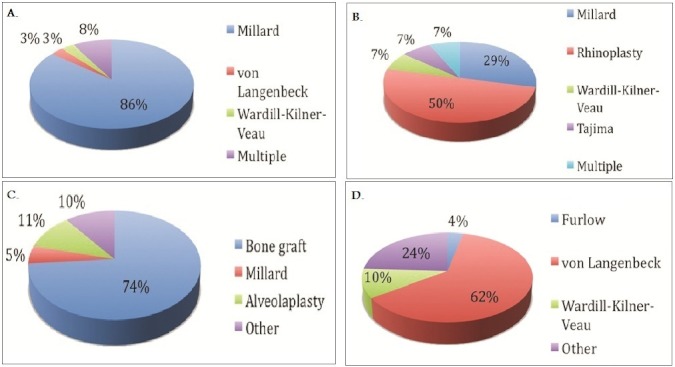
Protocols for repair of complete unilateral cleft lip and palate presenting at age 6 or greater for: A. cleft lip B. cleft nasal deformity C. alveolar deformity D. hard palate

**Figure 4:**
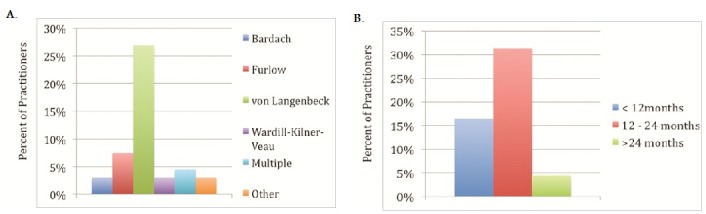
Practitioner's repair of isolated cleft palate presenting in the neonatal period A. Practitioners’ preferred technique B. Preferred age of repair

About 60% of practitioners report up to half of patients with cleft lip and palate present to their practice usually from birth to 6 years of age (Figure 1). This finding was similar in patients with isolated cleft palates. In the neonatal period, the lip deformity and nasal deformity in patients with complete unilateral cleft lip and palate was repaired in more than half of patients by 6 months of age (Figure 2). The timing of repair of those deformities contrasts with treatment of the alveolus deformity in the same group of patients. Treatment of the alveolus deformity exhibited a bimodal distribution, with most patients being treated at less than one year of age, or greater than 6 years of age (Figure 2). The majority of hard palate deformities in neonates were treated by 18 months of age (Figure 3). For isolated cleft palate patients presenting as neonates, the preferred age of treatment was within the first 24 months of life (Figure 4).

### Preferred Surgical Technique

In the neonatal period, the lip deformity in patients with complete unilateral cleft lip and palate were preferentially treated with Millard′s technique (Figure 2). The nasal deformity in the same group of patients was addressed by cleft rhinoplasty in approximately 12% of cases and nasoalveolar molding in 6% of patients, while the alveolus was addressed primarily by bone grafting (Figure 2). For management of the soft palate, the most commonly employed technique was von Langenbeck′s (26.9%), followed by Furlow′s palatoplasty (10.5%). Likewise, the main approach for treatment of the hard palate was von Langenbeck's technique (Figure 2). In patients with isolated cleft palate presenting as neonates, the technique of choice was von Langenbeck's (Figure 4).

Data regarding treatment protocols for complete unilateral cleft lip and palate in patients presenting at or after the age of 6 years was also collated. The results were similar to the group presenting in the neonatal period. Again, Millard's technique was the approach of choice for lip repair and cleft rhinoplasty for management of the cleft nasal deformity (Figure 3). The overwhelming majority of alveolar deformities (74%) were addressed by bone grafting (Figure 3). The soft palate was managed via von Langenbeck's approach in 41% of cases and by Furlow's palatoplasty in 26% of cases. The hard palate in this age group was mostly managed by von Langenbeck's technique in 62% of cases (Figure 3).

### Cleft Care Teams

**Figure 5:**
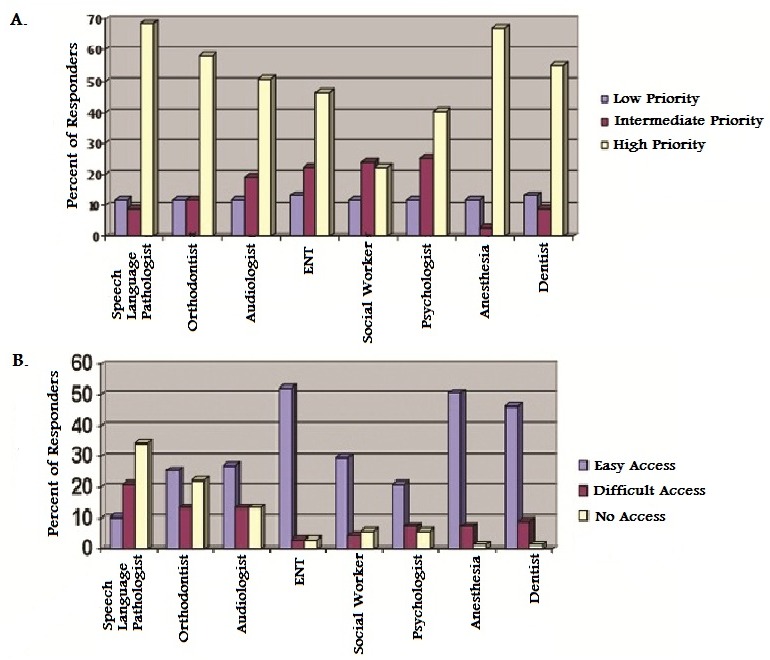
Practitioners’ evaluation of: A. Priority of access to cleft care services B. Ease of access to cleft care services

Information regarding the clinical situation of practicing providers, access to services, priority of services, and treatment challenges facing practitioners were explored. Practitioners were asked to rate the priority of access to various cleft care services. As shown in Figure 5, the core multidisciplinary composition critical to cleft care efforts were evaluated and responding practictioners rated all categories as high priority. Information regarding the ease of access to cleft care services is also reported. More than a third of practitioners had no access to speech language pathologists, and in cases where access was available, an additional 21% rated their access to this service as difficult. Similarly, 22% of practitioners had no access to orthodontists and 13% had no access to audiologists. The most easily accessible services were ENT (52%), safe anesthesia (51% ), social work (30%), psychology (21%), and dental services (46%) as shown in Figure 5.

### Cleft Care Treatment Challenges

**Figure 6:**
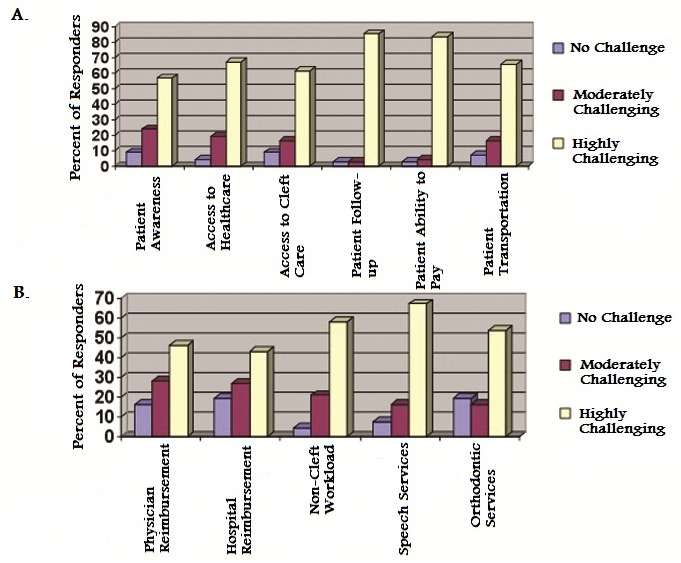
Practitioners’ evaluation of: A. Treatment challenges facing cleft care practitioners B. System-based challenges facing cleft care practitioners

Treatment challenges facing cleft care practitioners were investigated and reported on a Likert scale ranging from 1 (no challenge) to 5 (most challenging). This was then further classified into patient-related factors and physician/hospital-related factors. Patient-related factors affecting care included patients′ awareness, access to healthcare and cleft care, reliability of follow-up, availability of transportation, and the ability to pay. Across all the above categories, providers rated these factors as highly challenging for patients seeking cleft care (Figure 6). Physician-limiting factors rated as highly challenging to care delivery included hospital and physician reimbursements, volumes of non-cleft workload, and access to supporting services such as speech and orthodontics (Figure 6).

## Discussion

This study is the first report to our knowledge to delineate the treatment protocols employed for treatment of cleft lip and palate deformities by African cleft care providers. Millard′s technique was the most commonly employed approach for treatment of cleft lip deformities. Both the soft and hard palates were most commonly managed using von Langenbeck′s technique. Similarly, in patients with isolated cleft palates, the technique of choice was von Langenbeck′s approach, with the preferred age of treatment being within the first 24 months of life.

The second part of our analysis focused on constraints and barriers to cleft care encountered by practitioners. As detailed in the results, the data exhibits the plethora of factors that continue to pose formidable challenges to cleft care. These include both patient-related and physician-related factors. In the future, it would be beneficial to have a more concerted effort directed towards patient, family, and societal education regarding cleft awareness, treatment options, and the importance of regular follow-up. Difficulties with patient transportation are rampant, and oftentimes individuals with clefts are ostracized to the most remote areas where transportation access is poor and patients’ ability to work and earn the money necessary for care is severely limited. It is our hope that an increase in mission-oriented and more long-term cleft care teams would provide better access to patients located in more remote parts of the continent.

This study findings also emphasize the financial constraints, lack of access to concerted multidisciplinary teams, and the lack of necessary infrastructural amenities necessary to obtain and deliver cleft care. Practitioners reported physician and hospital reimbursement as key variables impacting care delivery. This implies there is either difficulty or lack of appropriate compensation mechanisms in place for practitioners and hospitals that are necessary to support sustainable cleft care programs. One cannot overemphasize the importance of integrating supporting cleft care teams to successful overall outcomes.

There are several isolated reports that have shed light on some of the factors constraining cleft care delivery. Mossey addressed several daunting challenges to cleft care in developing countries in his review of the World Health Organization report on the state of science in the field of craniofacial anomalies. Some of the challenges he highlighted were difficulties in attaining homogenous and adequate sample sizes for randomized trials, lack of long-term follow-up, cost of rehabilitation, and difficulties with access [11]. Pham and Tollefson in their retrospective review of cleft cases at the Harare Central Hospital reported contributing socioeconomic factors challenging cleft care [9]. The authors highlight the role of poverty, emigration of specialists to other countries, high prevalence of HIV, and decreased life expectancy as barriers to cleft care.

Donkor et al. noted an increasing number of cleft surgeries with each successive year over a five-year period in Kumasi, Ghana [12]. The authors attributed this finding to greater community awareness, free surgeries by local surgeons, and free operations by visiting surgical teams, again emphasizing that offering financial, training, and logistic support to cleft teams will help promote more surgeries for cleft patients. When Onah et al. compared their experiences from two West African sub-regional centers, they noted a high dropout rate after lip repair, and the subsequent difficultly of patient follow-up and compliance [13]. Experience with safe anesthesia in remote areas also presents its own set of challenges and Hodges et al. stressed this based on their reported experience [14]. In addition, the optimal environment to deliver cleft care is often not available. This is underscored in the report by Robin et al [15] in which the authors discuss management issues for children with clefts, drawing attention to the fact that the best environment for optimal cleft care is in a multidisciplinary team approach consisting of, but not limited to, audiology, speech pathology, otolaryngology, genetics, and occupational and feeding therapy. In an interesting correspondence to the editorial by Dupuis [16], Abernavoli [17] noted that partnership between local and visiting teams not only empowered the local medical community, but also enhanced the autonomy of local professionals. The author noted that the presence of overseas teams, when well integrated with local community teams, provided an avenue for both teams to develop initiatives to promote cleft care on a long-term basis.

Overall, this study elucidated important queries about surgical approaches for cleft lip and palate management and barriers to cleft care delivery in the region. However, there are some limitations to this study. One such limitation is that the majority of responders were from the West African region where the conference took place. Thus, it is likely that the data obtained is more representative of this region. Accessibility and ease of transportation may have played a role in this observed trend. Secondly, the total number of participants included medical students, dental technologists, social workers, nurses, and speech therapists. Of the 225 participants, only 140 were strictly direct practitioners. Fifty-four of the 68 responders were surgeons, resulting in lower overall response rates. Regardless of these potential limitations, this study provides significantly useful information about the region being studied.

## Conclusion

We feel that in addition to visiting surgical teams contributing to cleft care, provisions need to be made to address the financial, infrastructural, and socioeconomic barriers to cleft care in this region. By focusing on patient education, providing infrastructural support, and addressing socioeconomic hardship both at the institutional (physician) and individual (patient) levels, cleft care providers can partner with African cleft practitioners in a more dynamic, effectual, and valuable manner to promote cleft care in the region.

## Additional material

Anonymous Survey of Attendees of the Pan-African Congress on Cleft Lip and Palate Repair Feb. 4-7, 2007 (PDF 25K)
